# Rare and in Your Chair: An Emerging Use of Meropenem in a Case of Klebsiella pneumoniae Endophthalmitis Following Cataract Surgery

**DOI:** 10.7759/cureus.81977

**Published:** 2025-04-09

**Authors:** Shu Chyi Ong, Sruban Suparmaniam

**Affiliations:** 1 Ophthalmology, Hospital Teluk Intan, Teluk Intan, MYS

**Keywords:** acute blindness, cataract surgery, endophthalmitis, extended spectrum beta-lactamase (esbl), intravitreal injections, klebsiella pneumoniae, meropenem, multidrug-resistant organism (mdro), ophthalmology

## Abstract

Postoperative endophthalmitis is an infrequent but devastating complication that can happen after cataract surgery. The current mainstays of bacterial endophthalmitis treatment are intravitreal (IVT) antibiotics and vitrectomy. We hereby report a rare case of extended-spectrum beta-lactamase (ESBL)-producing *Klebsiella pneumoniae* in chronic postcataract surgery endophthalmitis, which was treated successfully with IVT and intravenous meropenem. An elderly female patient who underwent an eventful cataract surgery with anterior chamber intraocular lens (ACIOL) insertion in the right eye (RE) presented with profound painful vision loss. Clinical examination revealed a drop in her visual acuity and intense inflammation in the RE anterior chamber (AC). She was treated for chronic postcataract surgery endophthalmitis based on clinical examination. IVT tapping was performed, and IVT conventional antibiotics (vancomycin/ceftazidime) were given. Subsequently, her antibiotics were substituted with meropenem and given IVT and intravenously based on vitreous tapping culture and sensitivity. Following treatment, her vision improved on her RE. This case was reported to highlight the importance of high index of suspicion among clinicians for the possibility of antibiotic resistance, and the significance of substituting conventional antibiotics with a more susceptible one may yield better outcomes.

## Introduction

Postoperative endophthalmitis is a type of exogenous endophthalmitis. The average rate for postoperative endophthalmitis of cataract surgery in Malaysia from 2008 to 2014 was estimated to range from 0.06% to 0.11%; among cataract surgeries, phacoemulsification had the lowest percentage (0.07%), followed by extracapsular cataract extraction (ECCE) (0.10%), and other cataract surgery (phacoemulsification converted to ECCE and intracapsular cataract extraction (ICCE) (0.29%) [1]. New resistant bacterial strains have been identified and extended to the field of ophthalmology. The microbial spectrum seen in postoperative endophthalmitis depends on various factors, including environmental factors, apart from the types of surgery. Intravitreal (IVT) antibiotics are injected immediately within one hour of clinical diagnosis and are usually repeated after 48 to 72 hours [2]. The most commonly used regimens are vancomycin combined with ceftazidime or amikacin. Here, we describe the uncommon usage of IVT meropenem in a rare case of extended-spectrum beta-lactamase (ESBL)-producing *Klebsiella pneumoniae* in chronic postcataract surgery endophthalmitis.

## Case presentation

A 64-year-old Indian female patient, who was planned for a routine cataract surgery, underwent an eventful phacoemulsification converted to ECCE (posterior capsular rupture with vitreous loss) with anterior vitrectomy, surgical peripheral iridectomy, and angle-supported anterior chamber intraocular lens (ACIOL) implanted in the right eye (RE). Her wound was extended and sutured with 10/0 Nylon. Her subsequent reviews were normal until she presented to the ophthalmology clinic six months after surgery with a one-day history of acute painful vision loss. It was associated with a painful red eye, which was throbbing in nature. She denied any preceding causes like recent trauma or foreign body entry into her RE. Her presentation was not associated with any systemic symptoms like fever, headache, or generalized body weakness. No other relevant ocular or medical history was reported, and the patient was not taking any medication.

**Figure 1:**
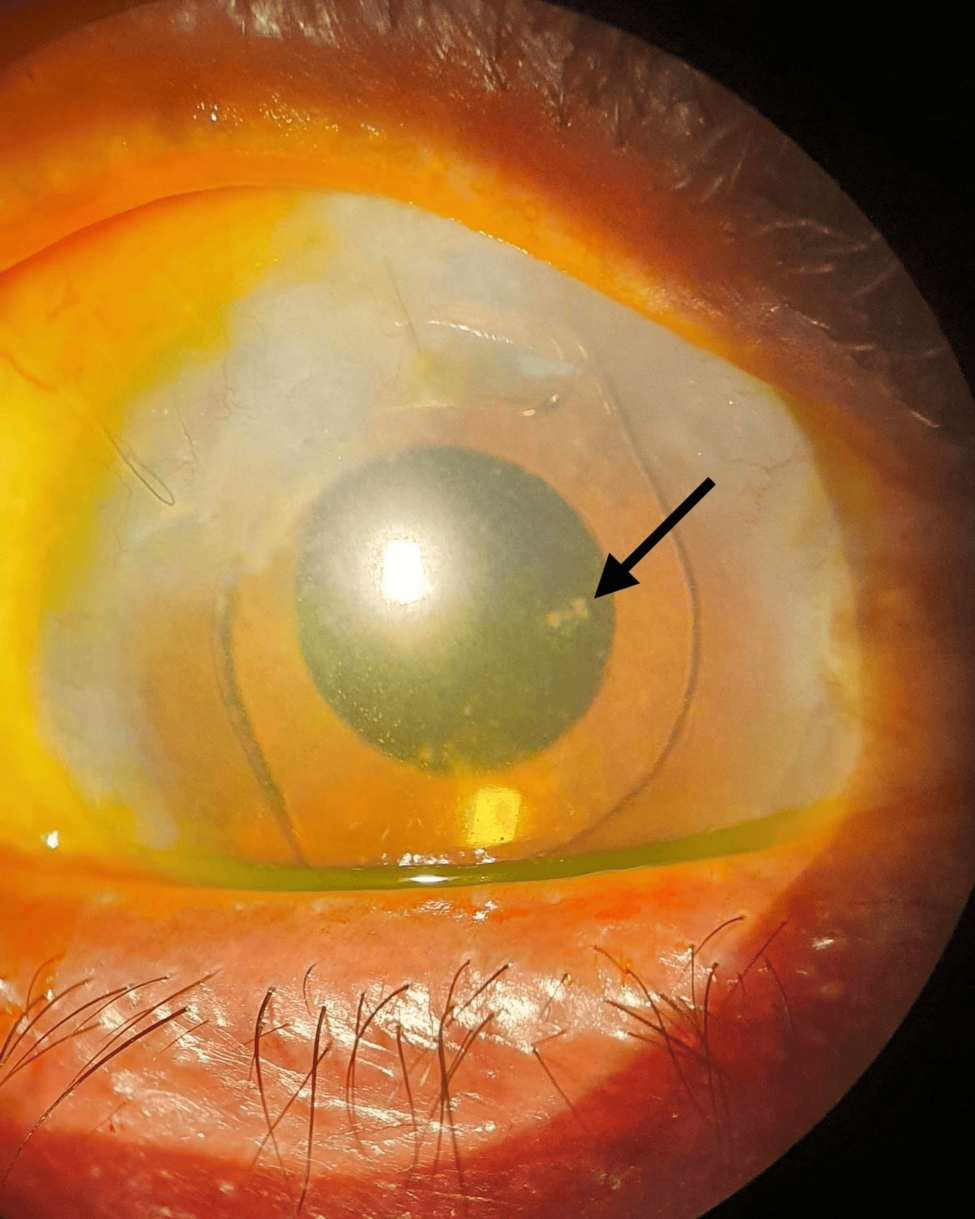
Right eye: ESBL Klebsiella pneumoniae endophthalmitis prior to intravitreal meropenem injection. Hazy cornea, intense anterior chamber inflammation with cells and fibrin, and multiple generalized keratic precipitates on the endothelium. Whitish deposits (black arrow) on the surface of anterior chamber intraocular lens ESBL: extended spectrum beta-lactamase

Her systemic examination was unremarkable. Her visual acuity on presentation was 6/36 in the RE and perception of light in the left eye (LE). The anterior segment examination on the RE showed conjunctival congestion, mild cornea haze, multiple whitish deposits on the surface of ACIOL, and the presence of intense inflammation in the anterior chamber (AC) (Figure 1), while her LE showed white cataract and no signs of inflammation. The intraocular pressures were within normal limits. No fundus view was observed on both eyes.

**Figure 2:**
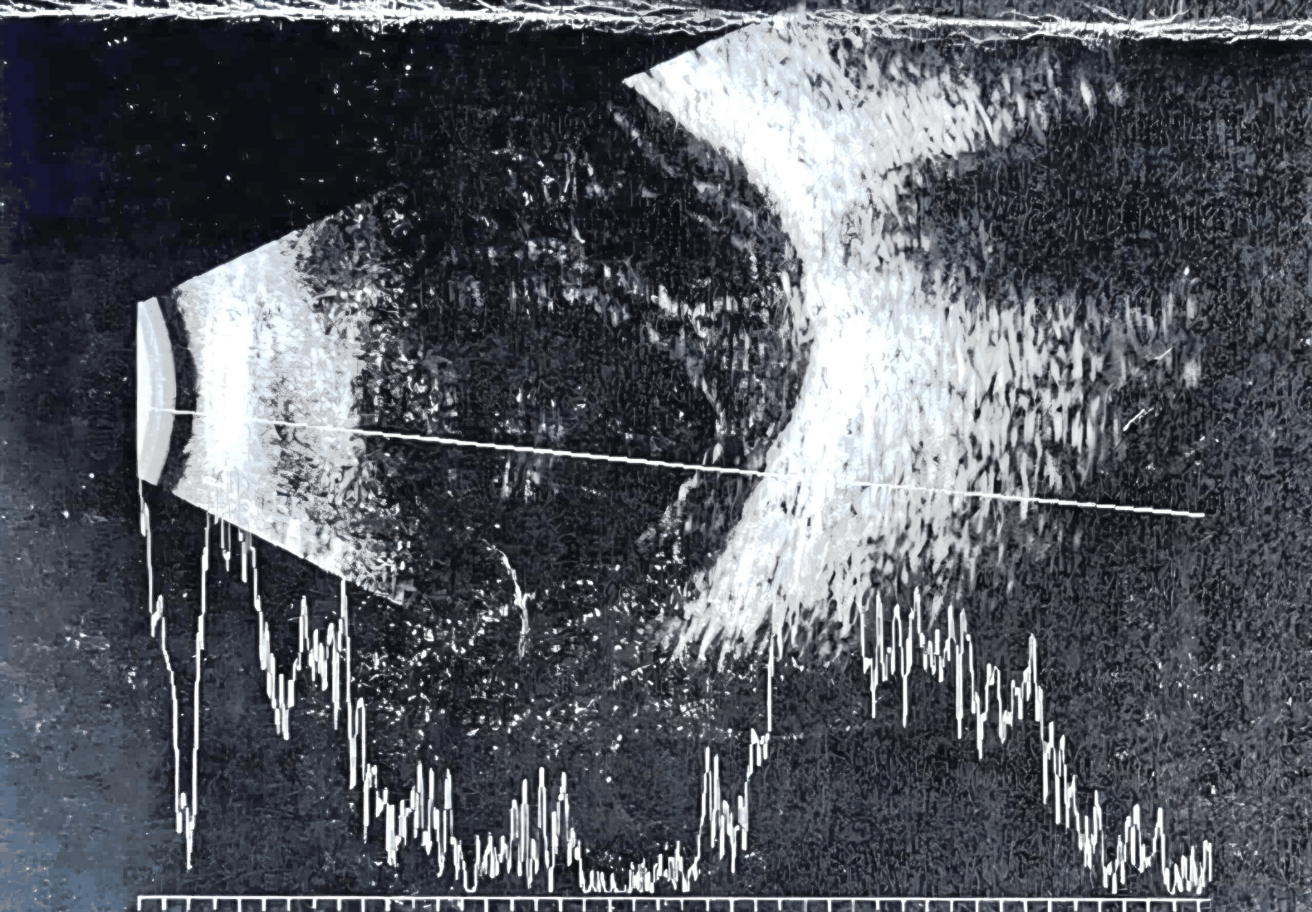
B-scan of the right eye showed vitreous opacities with loculations; retina flat B-scan: brightness scan ultrasonography

Brightness scan ultrasonography (B-scan) of the RE showed vitreous opacities with loculations and a flat retina (Figure 2) and normal findings on her LE. Based on these clinical findings, she was diagnosed with chronic postcataract surgery endophthalmitis. Hence, RE IVT tapping was commenced on the same day and portrayed a straw-colored vitreous. The vitreous specimen was sent for culture and sensitivity (C/S) and Gram stain. A combination of IVT vancomycin 2 mg/0.1 mL and ceftazidime 2 mg/0.1 mL injection was given. Ophthalmic and systemic treatment was initiated (guttae moxifloxacin 0.5% hourly, guttae atropine 1% once daily, guttae dexamethasone 0.1% every two-hourly intervals, and oral ciprofloxacin 750 mg twice daily), and the patient was admitted for further monitoring and treatment. She was given a repeated dose of IVT antibiotics (vancomycin/ceftazidime) after 48 hours; however, not much improvement was seen in her RE with her visual acuity of 6/36. She was also counselled regarding curative treatment, which may involve vitrectomy and complete removal of the capsular bag with IOL removal or exchange, but the patient was not in agreement with that option.

**Figure 3:**
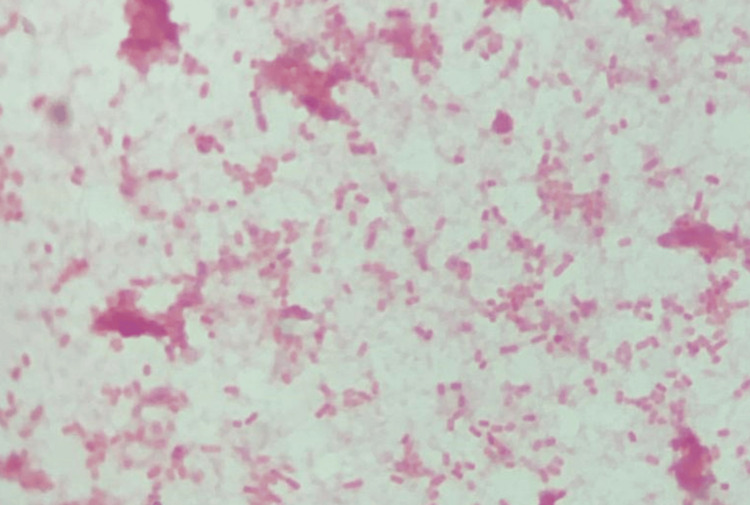
Gram staining shows Gram-negative rod-shaped bacilli

**Figure 4:**
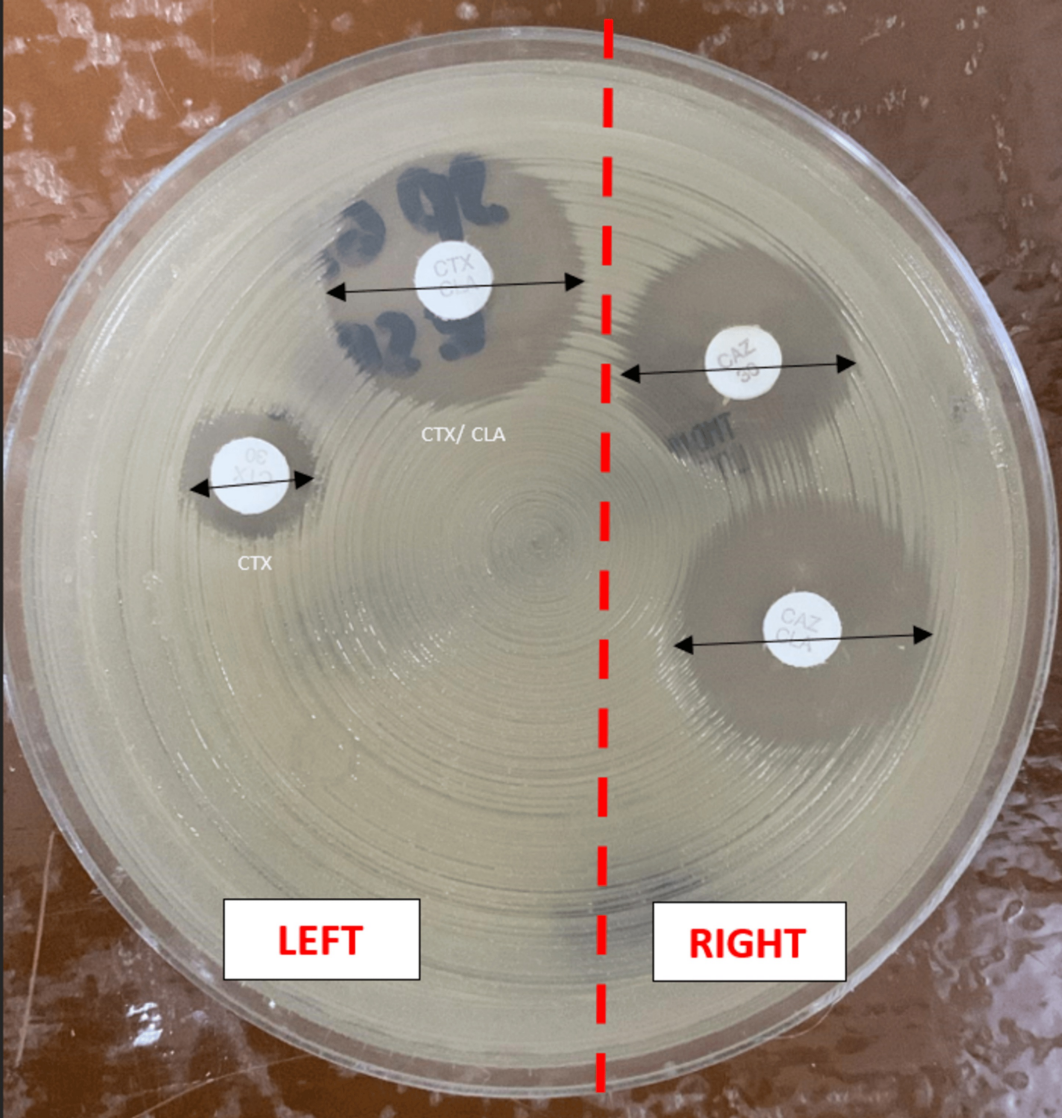
Kirby-Bauer test shows the antibiotics susceptibility testing using the combination disk method for ESBL detection. Zone diameter: ≥5 mm difference between the cefotaxime (CTX) and cefotaxime/clavulanic acid (CTX/CLA) disks on the left, and the ceftazidime (CAZ) and ceftazidime/clavulanic acid (CAZ/CLA) disks on the right, which suggests an ESBL-producing strain. * CTX/CLA-CTX = 22 mm-6 mm = 16mm (≥5 mm) * CAZ/CLA-CAZ = 22 mm-16 mm = 6 mm (≥5 mm) ESBL: extended-spectrum beta-lactamase

Her blood investigations (full blood picture, C-reactive protein, renal profile, urine full examination and microscopic examination, liver function test, blood C/S, urine C/S) showed normal findings and no growth in the cultures. The chest radiograph and abdominal ultrasound were performed to rule out an endogenous source, but both were normal. Vitreous Gram staining showed Gram-negative rod-shaped bacilli (Figure 3). The culture of vitreous C/S isolated (ESBL)-producing *Klebsiella pneumoniae* (Figure 4) was only sensitive to the carbapenem group while showing an extensive range of resistance to penicillins, cephalosporins, and aminoglycosides.

**Figure 5:**
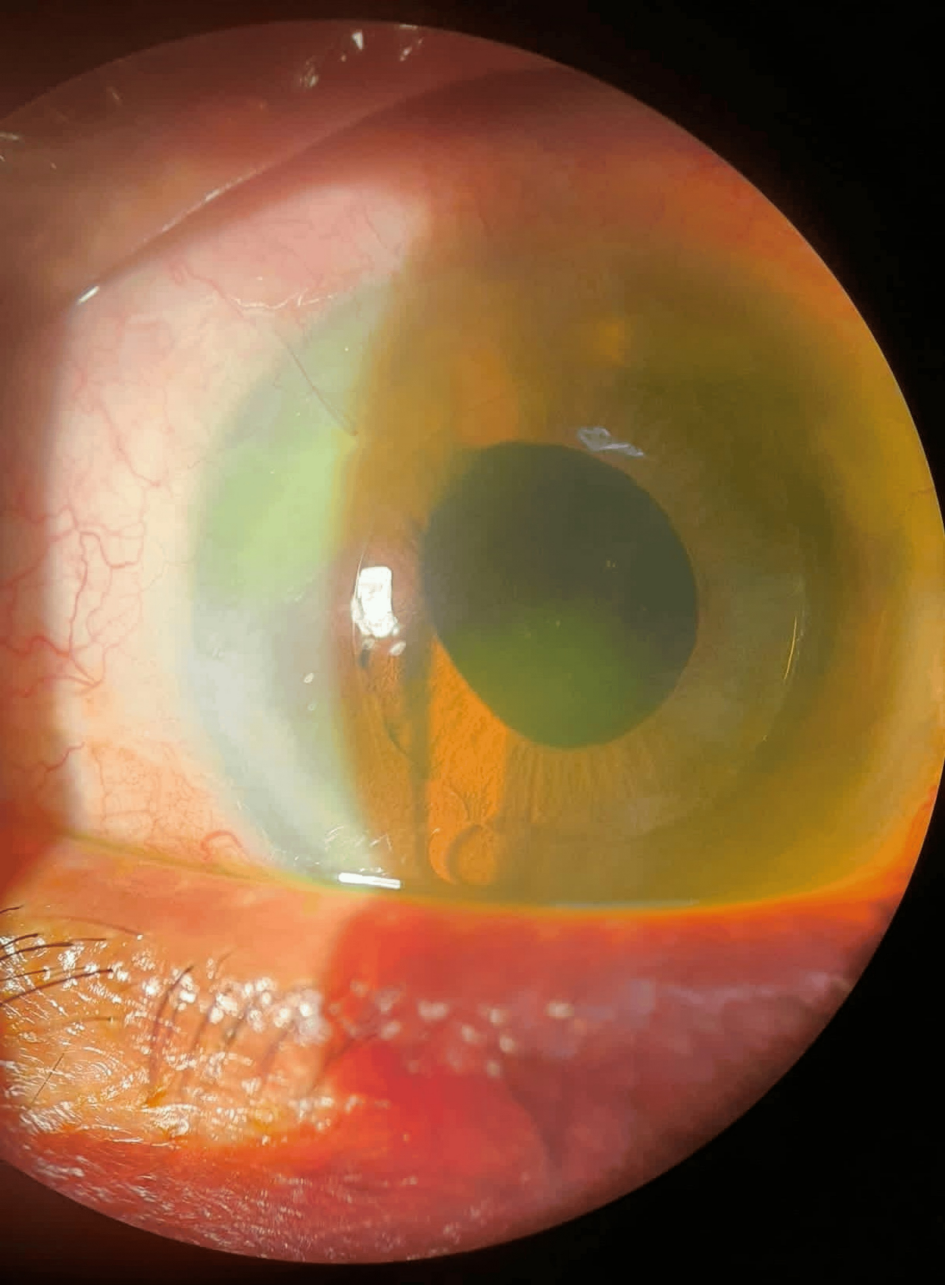
Right eye: significant improvement noted on day 7 post-intravitreal meropenem injection; cornea clear, resolved anterior chamber inflammation and whitish deposits on the surface of anterior chamber intraocular lens

After consulting with the infectious disease team, the antibiotic meropenem was initiated. She was given intravenous meropenem 1 g three times a day for a duration of one week, together with IVT meropenem (0.5 mg/0.1 ml) injection into her RE for a total of three doses (repeated every 48 to 72 hours). The patient showed significant clinical improvement, with gradual improvement of her visual acuity from 6/36 to 6/24 (after one dose of IVT meropenem) and subsequently improved to 6/12 (after the third dose of IVT meropenem) (Figure 5).

Despite that, the intraocular inflammation subsided after the treatment. Upon discharge, her final best-corrected visual acuity was 6/12. There were no AC cells seen; fundoscopy showed a pink optic disk with the presence of vitritis +1, vitreous clump inferiorly, and the macula showed epiretinal membrane. The whitish deposits on the surface of ACIOL also resolved. She was discharged home after two weeks of admission. Her subsequent follow-ups for two months at our eye clinic were uneventful. No signs of recurrence were seen. Unfortunately, she defaulted on her follow-up at our eye clinic in view of logistics issues.

## Discussion

Endophthalmitis refers to intraocular inflammation that affects the AC and vitreous cavity of the eye, as well as other nearby ocular tissues such as the cornea, sclera, choroid, or retina. According to the Endophthalmitis Vitrectomy Study (EVS), there are two types of postoperative endophthalmitis: acute, which happens within six weeks of cataract surgery, and chronic, which happens at or beyond six weeks. A single-center study found that the incidence rate of chronic postoperative endophthalmitis is much lower (0.017%) than that of acute onset [3]. Primary sources of infection are from the ocular surface and the adnexa, but consideration needs to be taken regarding contaminated agents or surgical equipment used perioperatively [4]. Vitreous communication (e.g., via a posterior capsular rupture or YAG capsulotomy), specific IOL implants, and diabetes are known risk factors [5]. Endophthalmitis is one of the most devastating diagnoses in ophthalmology. Although great advancements have been made in its management, the prognosis remains unfavorable (less than 50% of patients can achieve a final visual acuity of 6/12 or better. Even with advanced usage of disinfectants and antibiotic delivery methods, the prognosis is compromised by the occurrence of bacterial resistance as presented in this case [6].

*Propionibacterium acnes* (*P. acnes*) is the most common bacterial pathogen isolated in chronic postoperative cataract surgery endophthalmitis. This Gram‑positive anaerobic bacterium is commonly found in normal human skin flora. In view of the anaerobic environment between the posterior capsule and the IOL optic, *P. acnes* typically embeds there.* P. acnes *infection is clinically characterized by signs such as white plaque on the posterior capsule and the IOL, keratic precipitates, and chronic uveitis with or without the presence of hypopyon. In this case, the vitreous culture for *P. acnes* was negative [7].

According to the EVS, culture-positive endophthalmitis cases involving Gram-negative species were involved in 5.9% of cases [8]. *Klebsiella* species are Gram-negative, encapsulated, anaerobic bacteria that are commonly found in the nasopharyngeal and gastrointestinal tract. Although *Klebsiella pneumoniae* is highly prevalent in Eastern Asia, its frequency is rising globally [9]. Multidrug resistance is defined as acquired nonsusceptibility to at least one agent in three or more antimicrobial categories. It comprised 5.2% of culture-positive cases, and 78.6% of those isolates were Gram-negative bacteria [10]. Some of the factors that contribute to *Klebsiella pneumoniae* virulence are the existence of capsular K1/K2 serotypes, hypermucoviscosity, and the presence of the magA gene and the blaNDM-1 gene, which are carbapenemase β-lactamases. The first-line therapy for infections caused by multidrug-resistant *Klebsiella pneumoniae*, particularly ESBL producers, is carbapenems, which was implemented in this case [11].

This patient came to us six months later with granulomatous uveitis changes. There were large precipitates on the cornea and IOL surface. Curative treatment may involve vitrectomy and complete removal of the capsular bag with IOL removal or exchange, but the patient was not in agreement with that option. In vitro, *Klebsiella pneumoniae* elicits a proinflammatory response in the human retinal pigmented epithelium, causing rapid progression and a guarded prognosis in endophthalmitis [9]. In a case series, the visual prognosis of *Klebsiella pneumoniae* endophthalmitis was poor and can lead to the final outcome of phthisis and blindness [10].

Clinical diagnosis and management of chronic postoperative endophthalmitis can be challenging and a race against time as described in this case. It is hard to define a treatment regime or extrapolate the guideline set for acute postoperative endophthalmitis, given the indolent nature of the organism [5]. In this case, the treatment was modified due to the lack of improvement after commencing conventional IVT treatment twice, and once the sensitivity studies became available. Meropenem is a carbapenem antibiotic with a broad antibacterial spectrum of activity. A study showed that usage of meropenem in vitro on human retinal pigment epithelium has no cytotoxic effect [12]. The usage of meropenem is preferred compared to imipenem, which shows a rapid fourfold higher vitreous concentration and exceeds the breakpoint for relevant Gram-positive and Gram-negative organisms [2]. In this case, the dose of IVT meropenem (0.5 mg/0.1 ml) was given, which was similar to another case published [13]. Although the usage of systemic antibiotics in endophthalmitis is quite controversial, we did use intravenous meropenem as the study shows it exhibits favorable intraocular penetration [12].

Antimicrobial resistance is a worldwide concern that pertains to the prophylaxis and treatment of endophthalmitis. This microbial resistance can be related to the vast usage of antibiotics in intraoperative and postoperative ocular surgeries. Prolonged usage of the antibiotic levofloxacin after cataract surgery can result in levofloxacin-resistant ocular surface flora, and discontinuation can restore the sensitive flora after six to nine months [13]. To the best of our knowledge, this is the first case of exogenous endophthalmitis of chronic postcataract surgery following ESBL-producing *Klebsiella pneumoniae* infection, which was successfully treated with meropenem systemically and IVT. There was one similar case in acute postcataract surgery endophthalmitis, which was treated successfully with core vitrectomy and a single dose of IVT colistin injection combined with intravenous colistin and topical colistin eyedrops [11].

## Conclusions

Chronic postoperative endophthalmitis always poses a predicament for management and prognosis. Immediate recognition and a high index of suspicion are needed among ophthalmologists for the possibility of antibiotic resistance in the absence of improvement within the initial 48 to 72 hours, even in the absence of laboratory sensitivity results. Despite the broad usage and availability of antibiotics in this era, the consideration of switching conventional antibiotics to more susceptible ones is commonly delayed or not practiced, given the lack of evidence. This rare case highlighted the importance of timely intervention and the prompt act of substituting ceftazidime antibiotics with the more susceptible meropenem antibiotics, which yield significant visual recovery even in multidrug-resistant-positive culture cases. 
